# Application of X-ray image measurement in the early diagnosis of sports injury of ankle ligament

**DOI:** 10.12669/pjms.37.6-WIT.4841

**Published:** 2021

**Authors:** Shuqiao Meng, Wenxia Tong, Shanshan Han

**Affiliations:** 1 Shuqiao Meng, PhD. Physical Education College of Yangzhou University, Yangzhou, 225000, Jiangsu, China; 2 Wenxia Tong, PhD. Physical Education College of Yangzhou University, Yangzhou, 225000, Jiangsu, China; 3 Shanshan Han, PhD. Shangqiu University, School of Sport and Physical Education, Shangqiu, 476000, Henan, China

**Keywords:** X-ray, Lauge-Hansen classification, Weber classification, Ankle fracture, Sports injury

## Abstract

**Objectives:**

To study the value of X-ray analysis method of ankle fracture based on injury mechanism to improve the imaging diagnosis level of ankle fracture.

**Methods:**

This study was conducted from January 2016 to December 2019. It included 105 cases of fractures caused by sprained ankle joints. Their age was between 21-81 years with an average of 49.5 years, The traditional X-ray analysis method (Group-A) and the injury mechanism-based ankle fracture X-ray analysis method (Group-B) were used to analyze X-ray image data. Group-B also performed Weber classification and Lauge-Hansen classification on cases. Installment.

**Results:**

Of the 105 patients with ankle fractures, 97 patients in Group-B were able to make Lauge-Hansen classification. Of these 97 ankle fractures, 137 were found in Group-A, and 158 were found in Group-B. The wrong diagnosis of fracture in Group-A was 18%, and the wrong diagnosis of fracture in Group-B was 0.5%. There was a statistically significant difference between the two groups (P <0.05).

**Conclusion:**

The X-ray analysis method of ankle fractures based on injury mechanism can effectively improve the detection rate of hidden ankle fractures and high fibular fractures, and reduce the missed diagnosis, which is superior to the traditional X-ray analysis methods. At the same time, Weber classification, Lauge-Hansen classification and staging can be made for most cases, which is conducive to guiding clinical treatment.

## INTRODUCTION

At present X-ray plain film is the main diagnostic method for ankle fractures, but now the X-ray analysis method commonly used in China has more missed diagnosis of ankle fractures, and less attention is paid to ligament injury.^1^,^2^ If the CT scan layer thickness and other parameters are not selected properly, post-processing is not done properly, and minor fractures will also be missed.^3^,^4^ Minor fractures and displacements in the ankle joint and inferior tibiofibular joint can cause abnormal joint stress.^3^ However, the current X-ray plain film analysis method only observes whether there are fractures and joint space changes. There is no systematic analysis according to the injury mechanism. There are many missed diagnoses. The image report is incomplete and inaccurate.^5^,^6^

To analyze the fracture based on the mechanism of ankle injury, it is necessary to clarify the relationship between ligament injury and fracture: ligament injury is equivalent to fracture, and medial ligament (triangular ligament) injury is equivalent to medial ankle fracture.^7^,^8^ In this study, the traditional X-ray analysis and the injury mechanism-based ankle fracture X-ray analysis methods were used to analyze the X-ray image data of the ankle fracture.

## METHODS

A total of one hundred five adult patients with ankle joint fractures and plain radiographs and CT scans were included in this study performed from January 2016 to December 2019.

### Inclusion criteria

(1) At least one fracture of the medial malleolus, lateral malleolus, and posterior malleolus. (2) Swollen medial and lateral malleolus, clinically suspected fracture.

### Exclusion criteria

(1) Non-sprained fracture. (2) Vertical stress fracture. A total of 105 patients with ankle fractures were included, aged 21-81 years, with an average of 49.5 years. There were 54 males and 51 females.

This study selected four image attending physicians with nearly ten years of working experience and randomly divided them into Group-A and Group-B. Group-A used the ankle fracture analysis, that is, observe the X-ray plain film image including whether there is a fracture, fracture site, and whether there is widening of the joint space. Group-B analyzed the flow chart of the ankle joint X-ray analysis based on the aforementioned injury mechanism. For those who can make Weber classification, Lange-Hansen classification and staging, make classification and staging, and record the fracture site and ligament injury in case of a suspected fracture of a high fibula, add a radiograph of the medial side of the upper middle section of the tibia and fibula. Multi-row spiral CT cross-section and multi-planar reconstructed images were used as the “gold standard” to determine whether the diagnosis results of Group-A and B were missed diagnosis of fracture and dislocation. The inferior tibiofibular joint separation standard adopts the inferior tibiofibular gap> 6mm, and the 1cm above the tibiotalar joint is selected as the standard, and the diagnostic standard for the widening of the translucent gap of the medial malleolus uses the gap> 6mm.

### Principles of X-ray imaging measurement

Wavelet transform is a new branch of mathematics that has developed rapidly in the past decade.^9^ It introduces the concept of multi-scale. The wavelet function is a family of functions *J_a,b(x)_* generated by the base wavelet Ψ through stretching and translation:


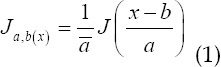


The wavelet transform of *f(x)∈L^2^(R)* is defined as an integration transform with the function family *J_a,b(x)_* as the integration kernel, as shown in the following equation:


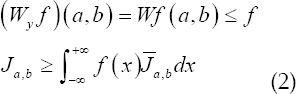


Among them, the base wavelet *J(x)* must meet the allowable conditions


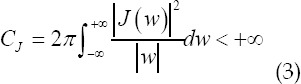


*a∈R^+^,b∈R,a,b* are scale factor and translation factor.

The two-dimensional discrete wavelet analysis used for image signal analysis is a direct derivation of one-dimensional analysis. Under the framework of multi-resolution analysis, through the Mallat tower algorithm, a fast algorithm for decomposition and reconstruction of discrete signals can be achieved. Let *n(x)* be a real wide stationary white noise with variance *e*^2^, and *W_n_(s,x)* be the wavelet transform whose scale is. *W_n_(s,x)* is also a random process, its variance is proportional to the square of wavelet norm ∥J∥


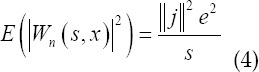


As the resolution decreases (s increases), the frequency also decreases, and the wavelet transform value of white noise gradually decreases, and the signal dominates; on the contrary, as the resolution increases (s decreases), the frequency It also gradually increased, the wavelet transform value of white noise also gradually increased, and the signal was overwhelmed by noise.

The filtering threshold of the CR system wavelet transform domain denoising process is based on two considerations: i) the reconstructed image has the same smoothness as the real image F; ii) the reconstructed image is the result of a minimum mean square error estimation. 10

The above two conditions require that the corresponding wavelet transform domain filter meet the following equations.


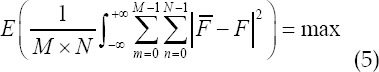



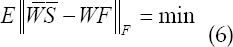


The above equations assume that the size of image *F* is pixels.

### SPSS19.0 analysis:

The comparison of fracture detection rate between the two groups was by χ^2^ test. The test level a = 0.05, P≤0.05 indicated statistical significance.

## RESULTS

The results of 105 cases of Weber classification: 30 cases of type A, accounting for 29%; 53 cases of type B, accounting for 51%; 11 cases of type C, accounting for 10%. 11 cases that couldn’t be classified by Weber accounted for 10%.

A hundred and five cases of Weber classification results were as follows. There were 30 cases of type A, accounting for 29%; 53 cases of type B, accounting for 51%; and 11 cases of type C, accounting for 10%. There were 11 cases that Weber could not classify, which accounted for 10%.

Lauge-Hansen classification results: 30 cases of supination and adduction, accounting for 31%, including 24 cases of stage one and six cases of Stage-II. There were 47 cases of pronation and external rotation, accounting for 48%, including 18 cases in Stage-II, seven cases in Stage-III, and 19 cases in Stage-IV. The majority of Stage-I is a combined injury of the anterior inferior tibiofibular ligament. X-rays have limitations on it and are difficult to diagnose. Six cases of pronation and external rotation type, accounting for 6%, including 5 cases of Stage-III and one case of Stage-IV. four cases of pronation and abduction type, accounting for 4%, were all Stage-III. Another 10 cases (11%) only saw a fracture of the medial malleolus or a widening of the translucent space of the medial malleolus, which was divided into pronation and external rotation type I or pronation and abduction type I, which couldn’t be further distinguished. Another 8 cases couldn’t be classified by Lauge-Hansen.

Among the 97 cases that could make Lauge-Hansen classification, 49 were male and 48 were female, with an average age of 49.5 years. Among these patients, the number of fractures found in the two groups is shown in Table-I, and the comparison of the fracture detection rates of the two groups is shown in Table-II. The fracture detection rates of the two groups are statistically different. The fracture detection rate of Group-B is higher than that of Group-A.

**Table-I:** Two group found the number of fractures.

	*Lateral ankle*	*Medial malleolus*	*Hind ankle*	*High fibula*	*Anterior tibial tubercle*	*total*
A	86	37	6	5	3	137
B	86	38	19	8	7	158

**Table-II:** Fracture detection rate between the two groups.

	*Number of fractures found*	*Number of fractures missed*	*total*	*Discovery rate (%)*
A	137	32	169	81.07
B	158	8	166	95.18
Total	295	39	335	88.06

## DISCUSSION

For Weber classification, types A, B, and C in this study accounted for 29%, 51%, and 10%, respectively, similar to those reported in the literature.^11^-^12^ In 8% of cases, Weber classification was not possible because there was no fibula fracture. 105 cases were all cases of ankle sprains, of which 97 cases were able to make Lauge-Hansen classification, and eight cases were not able to perform Lauge-Hansen classification. The types of injury in this study were: pronation and external rotation type accounted for 48%, pronation and adduction type accounted for 31%, pronation and external rotation type accounted for 6%, pronation and abduction type accounted for 4%, similar to those reported in the literature. Another 11% were divided into pronation and external rotation type I or pronation and abduction type I, because the performance of the two is the same and cannot be further distinguished.

According to related studies, the shape trend of the fracture line of the fracture is related to the effect of external force. According to the shape of the fracture line, it contributes to the type of external force [13]. The transverse fracture line is generally a traction fracture, oblique behavior push fracture, external rotation force generally leads to helical fracture, and the fracture line of the generalized fibula after supination and external rotation is laterally anterior to posterior.^14^,^15^ The pronation and external rotation type fibular fracture line is anterior to upper and lower. Most of the pronation and abduction type is oblique or partly transverse.^16^ The lateral fibula, that is, the pressure side, sometimes sees bone fragments.^17^ It is also helpful for the direction of some fracture lines that are difficult to type.

X-ray plain film can be used for Weber classification, Lauge-Hanse and classification injury of ankle fractures. In the staging, the injury is used as the starting point in a clockwise direction, and each stage cannot be skipped.^18^,^19^ Weber classification pays great attention to the joint injury of the lower tibiofibular ligament, but does not describe the injury of the medial malleolus and posterior malleolus.^20^ The medial malleolus and triangular ligament play an important role in the stability of the ankle joint, and posterior malleolus fractures account for 25% of the articular surface can be used as an indication for surgery. If the two are combined, the Lauge-Hanse classification of ankle fractures is simple, such as Weber Type fracture, it is easy to connect the Lauge-Hanse classification of the internal rotation and external rotation type, and then do the next analysis, and the analysis results will be reflected in the report in the next step, which will provide more information to the clinician, for example Fig.2, X-ray plain film analysis result is finally Weber, post-spin external rotation Stage-IV fracture, unstable fracture.

A study of the X-ray analysis method of ankle fractures based on the injury mechanism concluded that the use of Weber classification, Lauge-Hansen classification, and stage X-ray analysis of ankle fractures can effectively improve the detection of hidden ankle fractures and high fibular fractures, reduce missed diagnosis, and is superior to traditional X-ray analysis method. It is meaningful to improve the diagnosis level of ankle fractures by radiologists. Weber classification, Lauge-Hansen classification and staging can be made for most cases, which is conducive to guiding clinical treatment.

## CONCLUSIONS

X-ray plain film is the easiest and most economical method for diagnosis of ankle injury. Using the X-ray analysis method of ankle fracture based on injury mechanism, the diagnosis flow chart was summarized, and the X-ray diagnosis thinking of ankle fracture was clarified. By using two different X-ray image analysis methods to analyze 105 patients with ankle sprains, it was found that the method introduced in this study can effectively reduce the chances of missed diagnosis rate and improve the diagnosis level of ankle fractures. Ninety-seven patients with ankle joint injuries were classified by Weber, Lauge-Hansen classification and staging to guide clinical treatment.

### Authors Contribution:

**SM:** Conceived the study, literature review, participated in its design, coordination, analyzed the data, helped to draft the manuscript, is responsible and accountable for the accuracy or integrity of the work.

**SH:** Helped in design, data collection, article drafting & critical revision.

**WT:** Takes the responsibility and is accountable for all aspects of the work in ensuring that questions related to the accuracy or integrity of any part of the work are appropriately investigated and resolved.
